# Cross-Validation of the Spanish HP-Version of the Jefferson Scale of Empathy Confirmed with Some Cross-Cultural Differences

**DOI:** 10.3389/fpsyg.2016.01002

**Published:** 2016-07-12

**Authors:** Adelina Alcorta-Garza, Montserrat San-Martín, Roberto Delgado-Bolton, Jorge Soler-González, Helena Roig, Luis Vivanco

**Affiliations:** ^1^Service of Oncology, University Hospital Jose E. Gonzalez – Autonomous University of Nuevo LeónMonterrey, Mexico; ^2^Scientific Computing GroupLogroño, Spain; ^3^Ibero-American University FoundationBarcelona, Spain; ^4^Hospital San PedroLogroño, Spain; ^5^Center for Biomedical Research of La RiojaLogroño, Spain; ^6^Department of Medicine (Gesec and Gerds Group), Faculty of Medicine, University of LleidaLleida, Spain; ^7^Borja Institute of Bioethics, Ramon Llull UniversityBarcelona, Spain; ^8^National Centre of Documentation on BioethicsLogroño, Spain

**Keywords:** empathy, physician, cross-culture comparison, Spanish, psychometrics

## Abstract

**Context:** Medical educators agree that empathy is essential for physicians' professionalism. The Health Professional Version of the Jefferson Scale of Empathy (JSE-HP) was developed in response to a need for a psychometrically sound instrument to measure empathy in the context of patient care. Although extensive support for its validity and reliability is available, the authors recognize the necessity to examine psychometrics of the JSE-HP in different socio-cultural contexts to assure the psychometric soundness of this instrument. The first aim of this study was to confirm its psychometric properties in the cross-cultural context of Spain and Latin American countries. The second aim was to measure the influence of social and cultural factors on the development of medical empathy in health practitioners.

**Methods:** The original English version of the JSE-HP was translated into International Spanish using back-translation procedures. The Spanish version of the JSE-HP was administered to 896 physicians from Spain and 13 Latin American countries. Data were subjected to exploratory factor analysis using principal component analysis (PCA) with oblique rotation (promax) to allow for correlation among the resulting factors, followed by a second analysis, using confirmatory factor analysis (CFA). Two theoretical models, one based on the English JSE-HP and another on the first Spanish student version of the JSE (JSE-S), were tested. Demographic variables were compared using group comparisons.

**Results:** A total of 715 (80%) surveys were returned fully completed. Cronbach's alpha coefficient of the JSE for the entire sample was 0.84. The psychometric properties of the Spanish JSE-HP matched those of the original English JSE-HP. However, the Spanish JSE-S model proved more appropriate than the original English model for the sample in this study. Group comparisons among physicians classified by gender, medical specialties, cultural and cross-cultural backgrounds yielded statistically significant differences (*p* < 0.001).

**Conclusions:** The findings support the underlying factor structure of the Jefferson Scale of Empathy (JSE). The results reveal the importance of culture in the development of medical empathy. The cross-cultural differences described could open gates for further lines of medical education research.

## Introduction

Medical empathy is defined as a predominantly cognitive (rather than emotional) attribute that involves the ability to understand (rather than feel) patient's experiences, concerns and perspectives, and communicate this understanding (Hojat et al., 2002). Empathy has been listed consistently as one of the key elements of professionalism. The importance of empathy, as key element of professionalism, has been discussed in medical education and health care research (Veloski and Hojat, 2006), and in global bioethics (Vivanco and Delgado-Bolton, 2015).

Despite its importance in enhancing these relationships and improving patient care, research on physician empathy has been limited for two main reasons. Firstly, the theoretical investigation of physician empathy has been hampered by ambiguity in its conceptualization and definition. Secondly, empirical research in this area has been limited by a lack of tools to gauge the empathy of medical students and physicians. Nevertheless, the development of standardized instruments currently allows assessing the empathy in the interactions that take place in the context of healthcare (Hemmerdinger et al., 2007). One of the most popular instruments for this purpose is the Jefferson Scale of Empathy (JSE). Medical education researchers of the Jefferson Medical College, in the United States, developed this tool. The generic version of the scale was originally developed to measure medical students' orientations or attitudes toward empathic relationships in the context of patient care (Hojat et al., 2001). However, very soon there was a demand to use the scale for administration not only to medical students, but also to physicians and other health professionals involved in patient care, and all health professions students other than medical students. Thus, the authors decided to slightly modify the content of the generic scale so that three versions would be available (Hojat, 2006): one version for administration to medical students (the S-Version); a second version for administration to physicians and other practicing healthcare professionals (the HP-Version); and the third version for administration to students of all healthcare professions other than medical students (the HPS-Version).

Construct validity refers to the extent to which a test measures the theoretical constructs of the attribute that it aims to measure. In this sense, factor analysis of the JSE helps to determine whether the underlying factors of the scale are consistent with the theoretical constructs of the concept measured, being in this case empathy. Following this principle, the factor analysis of the generic scale of the JSE revealed four preliminary factors, which were consistent with the multifaceted concept of empathy reported in the literature (Spiro et al., 1993). The first of those factors included 10 items. This factor was called “the physician's view of the patient's perspective.” The second factor included five items, and it was called “understanding patient's experiences.” The third factor, composed by two items, was called “ignoring emotions in patient care” (that refers to the opposite pole of standing in a patient's shoes). Finally, the fourth factor, composed by two items, was called “thinking like the patient.” Following the recommendation of a minimum number of three items per factor (Velicer and Fava, 1998), the authors considered the last two factors had less stable factor pattern than the first two. Subsequent analysis showed that the first factor was the most salient among all other extracted factors.

The factor analysis of the HP-Version of the JSE showed three definitive underlying factors:

- A first major factor that is composed by 10 positively worded items. This factor, described as the core cognitive ingredient of the empathy and as the stepping-stone in empathic engagement, was called “perspective taking.” It is similar to the first factor described in the generic version.
- A second factor that is composed by eight negatively worded items. This factor was called “compassionate care.” According with the authors, this construct is conceptually similar to the two factors that emerged in the generic version: “emotions in patients care” and “understanding patient's experiences.”
- Finally, a third factor that is composed by two other negatively worded items. This factor was called “standing/walking in the patient's shoes” (the positive pole of the contents of the negatively worded but reverse-scored items). This last factor was described as a trivial component of the JSE, and similar to the factor “thinking like the patient” of the generic version.

According to the authors, these findings suggest that the factor structure of the JSE is consistent with the notion of the multidimensionality of empathy (Davis, 1983; Kunyk and Olson, 2001). In addition, the stability and the similarity between the factor structure and components across different samples (students and professionals) and across different versions of the scale provide, according to the authors, further support for the JSE's validity (Hojat, 2016).

Since its creation, both researchers and medical educators at international level have acknowledged the validity of the JSE. Its first cross-cultural adaptation was designed by Mexican researchers who administered the S-Version to medical students (Alcorta-Garza et al., 2005). Subsequently, the JSE was translated into 42 languages and is currently used worldwide in 60 countries located in Europe, the Middle East, Africa, Asia, North America, Latin America, and New Zealand (Hojat et al., 2011a). In order to improve the clarity of the scale for an international audience, minor revisions were made in the wording of verbatim translation of some items that created some confusion in Italian and Spanish translations (Hojat, 2016). This is the case of the item 18: “I do not allow myself to be touched by intense emotional relationships between my patients and their family members” (a negatively worded item),” in the generic version. The symbolic meaning of “to be touched by” (to be affected or emotionally stirred) was not apparent in the translated versions. Therefore, the authors decided to replace “to be touched” by “to be influenced” (Hojat, 2016).

However, it is difficult to say whether due to these changes, to some unresolved translation issues, or due to cultural differences, the factorial position of this item remains still problematic in the factor analysis of some translations (Alcorta-Garza et al., 2005; Magalhaes et al., 2011; Tavakol et al., 2011b; Paro et al., 2012; Shariat and Habibi, 2013; Wen et al., 2013; Leombruni et al., 2014). Despite this issue, most of the studies using the JSE conducted in different countries report evidence supporting construct validity, criterion-related validity, predictive validity, internal consistency reliability, and test-retest reliability. In most of the cases, exploratory factor analysis using principal component analysis (PCA) with orthogonal rotation was used to determine the factor structure of the JSE. Exploratory factor analysis studies have often resulted in the three aforementioned factors (Alcorta-Garza et al., 2005; Paro et al., 2012; Wen et al., 2013). There are only a few adaptations were the factor structure of the JSE was studied using confirmatory factor analysis (CFA). In some cases, CFA was used to confirm a factor structure resulting from a previous PCA (Magalhaes et al., 2011; Tavakol et al., 2011b), and in others to confirm whether if the sample studied fitted the original theoretical model (Shariat and Habibi, 2013; Leombruni et al., 2014).

All these studies provide clues about the underlying components of the JSE, not only in samples from different disciplines, but also in a wide variety of cultural contexts. However, despite cumulative evidence, in a recent publication some of authors recognize the need to undertake additional research using samples from different professional and cultural contexts (Hojat and Lanoue, 2014).

Spanish is the second most widely spoken language in the world in terms of native speakers after Chinese. It is the official language of more than 20 countries, most of them in Latin America (Otero and Powell-Davies, 2011), which is where almost 90% of the population of native Spanish speakers lives. However, its different varieties along this territory involve significant cultural differences (Mato, 2008), which are even more noticeable when compared to the variety spoken in Spain (Oesterreicher, 2013). Conversely, Spain's cross-cultural diversity is higher because of constant migratory flows. This is also reflected in the structure of the Spanish Healthcare System, with one of the highest levels of cultural diversity in the European area (Sánchez-Sagrado, 2013).

Both the cultural diversity resulting from the language, and the cross-cultural characteristics of Spain and Latin America, provide an ideal scenario to test the psychometric properties of the JSE (Delgado-Bolton et al., 2015). In addition, a better understanding of the role of culture in the development of communication skills, professional behaviors, and lifelong learning abilities of health care practitioners is fundamental for the improvement of medical education, health management, and bioethics from a global scope (Vivanco and Delgado-Bolton, 2015).

This study has three purposes: to develop a validated translation of the JSE (HP-Version) that may be used by Spanish and Latin American researchers; to confirm the psychometric properties of the JSE in the Spanish language context; and to achieve a better understanding of the role of culture in the development of the medical empathy.

## Materials and methods

### Participants

The study is based on a sample of 896 healthcare professionals (physicians and physicians-in-training) involved in direct patient care in 13 healthcare institutions from Spain, Mexico, Colombia, Bolivia, and Argentina, who were invited to participate voluntarily and anonymously.

### Instrument

The participants completed the JSE (HP-Version). This questionnaire is a psychometrically sound instrument developed specifically to measure physicians' empathetic orientation in the context of patient care. The JSE includes 20 items, each answered on a 7-point Likert-type scale (1 = strongly disagree, 7 = strongly agree). Possible scores range from 20 to 140 and the higher the score, the greater the empathic orientation. The JSE identifies three factors: “perspective taking,” “compassionate care,” and “standing/walking in the patient's shoes” (Hojat, 2016).

### Complementary information

Information about age, gender, professional status, medical specialty, country of birth, country of studies, and country of current residence was collected through a complementary survey.

### Procedures

The original version of the JSE was translated into international Spanish, adapted, and reviewed using a cross-cultural back-translation procedure (Geisinger, 1994). Between 2014 and 2015, the translated version was administered to physicians and physicians-in-training from 13 institutions. The questionnaires consisted of paper forms provided together with an information letter in enclosed envelopes that were returned to the local researchers following a general protocol previously approved by an Independent Ethics Committee (Ref. CEICLAR PI 199). The work was carried out in accordance with the Declaration of Helsinki. There was no potential risk for participants, and anonymity was guaranteed throughout the process.

### Statistical assessment

Internal consistency reliability was calculated using Cronbach's alpha coefficient. Following the guidelines suggested by the American Educational Research Association, values higher than 0.7 were considered satisfactory.

To consider the underlying factors, the data obtained for the 20 items of the JSE were subjected to exploratory factor analysis. The purpose of this was to explore the association between the observed variables (items) and the latent variables (factors) using PCA with oblique rotation (promax) to allow for correlations among the extracted factors. The retained factors were limited to three so that the findings could be compared to the previously reported results of factor analysis (Hojat et al., 2002; Hojat and Lanoue, 2014). Retained factors were considered satisfactory when their eigenvalues were greater than one (Henson and Roberts, 2006).

The aim of the CFA tests was to discover whether if the observed data fitted a previously postulated model. In this study, the agreement of two models was tested using a CFA. In this regard, as opposed to a theory-generating model such as PCA, CFA is a theory-testing model that begins with a hypothesis prior to the analysis (Brown, 2014). This hypothesis can be based on theory, research, or both (Suhr, 2005). The first model tested in this study, Model A, is based on the 3-factor structure of the original HP-Version of the JSE, with American physicians (Hojat et al., 2002). The second model, Model B, is based on the 3-factor structure of the Spanish validated S-Version of the JSE, with Mexican students (Alcorta-Garza et al., 2005). The only difference between both models is the factor distribution of worded item 18: in Model A it is included in Factor 2 (compassionate care), whereas in Model B, it appears in Factor 3 (standing/walking in the patient's shoes). A third model, artificial Model AB, is based on a two-factor distribution hypothesis (Factors 2 and 3) for worded item 18, which was tested conducting preliminary CFA. This preliminary analysis was also used to test whether the underlying factors should be treated as correlated or uncorrelated.

*Robust WLS* is an estimation method used for structural equation modeling with ordinal observed variables with non-normality extremes /*Asymmetry*/≫ 3 and Kurtosis>8 (Muthén et al., 1997). Since the nature of the data meets these criteria, this was the estimation method used for the CFA. The goodness of fit indexes calculated to assess each model's fit were χ^*2*^ statistics and its subsequent ratio with degrees of freedom (χ^*2*^∕*df*), Comparative Fit Index (CFI), Tucker-Lewis Index (TLI), Root Mean Square Error of Approximation (RMSEA), and Standardized Root Mean square Residual (SRMR) (Muthén et al., 1997; Kline, 2005).

Group comparisons of empathy scores were performed. Gender, professional status (physicians and physicians-in-training), place of birth (Latin America and Spain), place of professional studies (Latin America and Spain), and residence (Latin America and Spain) were treated as dichotomous variables. Medical specialities were divided into the following groups: “non-hospital speciality” (this group included family medicine and occupational medicine specialties), “hospital speciality,” “medical-surgical specialty,” “surgical speciality,” and “other specialities.” For physicians without specialization, a “no speciality” group was created for non-specialist physicians. According to their migratory condition, physicians were divided into three groups: “Spaniards living in Spain,” “Latin Americans living in Spain,” and “Latin Americans living in Latin America.”

All analyses were performed using R statistical software, version 3.1.1 for Windows. The statistical analyses of the data also included multilevel (Bliese, 2013), nortest (Gross, 2012), and lavaan (Rosseel, 2012) packages.

## Results

Of the 896 participants who received the JSE, 715 were returned fully completed, giving an overall effective response rate of 80%. This response rate was higher than the minimum recommended to ensure the representativeness of the sample for mailed surveys to professionals (Gough and Hall, 1977).

The mean age was 35 years old with a 24–71 year-old age range (*SD* = 10.8). Three hundred and fifty-one (48%) of the physicians reported to be born in Spain and 347 (47%) of the physicians were born in Latin America. Thirteen countries were reported in this group (Mexico, Colombia, Bolivia, Argentina, Dominican Republic, Venezuela, Peru, Ecuador, Chile, Honduras, Cuba, El Salvador, and Uruguay). Seventeen physicians (2%) were born in non-Spanish-speaking territories. Eleven countries were reported in this group (Brazil, Italy, Ukraine, Morocco, Andorra, Belgium, Canada, France, Haiti, Moldova, and Ruanda). Finally, 18 (3%) physicians did not specify their country of birth.

The empathy score distribution, descriptive statistics, and reliability for the JSE in this study are described in Table 1.

**Table 1:** **Descriptive statistics and psychometric properties of the Spanish JSE-HP version**.

**Statistics**	**Value**
N	715
Possible range	20–140
Actual range	59–140
Mean	116
Standard deviation	14
**PERCENTILE**	
25th	108
50th (Median)	119
75th	127
**CRONBACH'S ALPHA COEFFICIENT**	
Entire group	0.84
Physicians	0.83
Physicians-in-training	0.84

### Components of the JSE

The three meaningful factors yielded by PCA had eigenvalues >1, a result that is in accordance with the factor structure described for the original version. The first factor, which reflected the original first factor, “perspective taking,” included 10 items with factor loadings higher than 0.30, accounting for 15.5% of the total variance. The second factor, which reflected the original second factor “compassionate care,” included seven items (one less than the original English version) with factor loadings higher than 0.30, accounting for 11.2% of the total variance. The third factor, which reflected the original third factor “standing/walking in the patient's shoes,” included two items with factor loadings higher than 0.30, accounting for 5.9% of the total variance. Worded item 18 (originally associated with Factor 2 in the English version) showed a low factor loading (0.24), associated with Factor 3.

A preliminary CFA revealed a good data fit for correlated Model AB. All items, with the exception of worded item 18, were significant for the three underlying factors (*p* < 0.001). Item 18 was significant for Factor 3 (*p* = 0.005), but not for Factor 2 (*p* = 0.075). Uncorrelated Model AB revealed poor data fit (ratio χ^2^/*df* > 13, *CFI* = 0.57, *TLI* = 0.52, *RMSEA* = 0.13, and *SRMR* = 0.14). Goodness of fit indexes for the correlated model AB, the correlated model A, and the correlated model B revealed good data fit for all cases. However, the item 18 was not statistically significant (*p* = 0.075) in factor 2 of model AB. Goodness of fit indexes for the three correlated 3-factor models, including *p*-values for item 18, are reported in Table 2.

**Table 2:** **Goodness of fit indexes for the three correlated 3-factor models of the Spanish JSE-HP version including *p*-values for item 18**.

**Model**	**χ^2^**	***df***	**Ratio χ^2^/*df***	**CFI**	**TLI**	**RMSEA**	**SRMR**	**Item 18**	
								**F2**	**F3**
Model AB	236	166	1.422	0.984	0.982	0.025	0.046	0.075	0.005
Model A	244	167	1.462	0.984	0.982	0.025	0.048	0.000	–
Model B	251	167	1.508	0.983	0.981	0.027	0.048	–	0.000

*χ^2^, Chi-square statistic; df, degrees of freedom; CFI, comparative fit index; TLI, Tucker-Lewis index; RMSEA, root mean square error of approximation; SRMR, standardized root mean square residual*.

Based on these findings, a final factor structure of the JSE is reported in Table 3. Goodness of fit indexes of this factor structure model was tested according to gender, professional status, place of birth, place of studies, and residence. The report of this analysis is shown in Table 4.

**Table 3:** **Items' measures and factor structure of the Spanish JSE-HP version**.

**Item**	**Statement**	**M (SD)**	**PCA^b^**	**CFA^c^**	***r^d^***
**FACTOR 1: “PERSPECTIVE TAKING”**					
2	My patients feel better when I understand their feelings	6.4 (1.1)	0.56	0.67	0.55
4	I consider understanding my patients' body language as important as verbal communication in caregiver-patient relationships	6.3 (1.1)	0.34	0.55	0.51
5	I have a good sense of humor that I think contributes to a better clinical outcome	5.4 (1.3)	0.46	0.49	0.34
9	I try to imagine myself in my patients' shoes when providing care to them	5.7 (1.4)	0.58	0.79	0.59
10	My patients value my understanding of their feelings which is therapeutic in its own right	5.7 (1.3)	0.59	0.76	0.51
13	I try to understand what is going on in my patients' minds by paying attention to their non-verbal cues and body language	5.9 (1.3)	0.57	0.83	0.59
15	Empathy is a therapeutic skill without which my success in treatment is limited	6.0 (1.4)	0.55	0.72	0.66
16	An important component of the relationship with my patients is my understanding of their emotional status, as well as that of their families	6.1 (1.2)	0.68	0.86	0.66
17	I try to think like my patients in order to render better care	5.4 (1.4)	0.53	0.57	0.45
20	I believe that empathy is an important therapeutic factor in medical or surgical treatment	6.4 (1.0)	0.61	0.63	0.58
**FACTOR 2: “COMPASSIONATE CARE”**					
1	My understanding of how my patients and their families feel does not influence my medical or surgical treatment ^a^	5.9 (1.9)	0.38	0.66	0.50
7	I try not to pay attention to my patients' emotions in history taking ^a^	5.8 (1.6)	0.34	0.82	0.56
8	Attentiveness to my patients' personal experiences does not influence treatment outcomes ^a^	5.8 (1.7)	0.57	1.16	0.67
11	Patients' illnesses can be cured only by medical or surgical treatment; therefore, emotional ties to my patients do not have a significant influence on medical or surgical outcomes^a^	6.1 (1.3)	0.66	0.94	0.61
12	Asking patients about what is happening in their personal lives is not helpful in understanding their physical complaints^a^	5.8 (1.8)	0.48	0.92	0.58
14	I believe that emotion has no place in the treatment of medical illness^a^	6.3 (1.4)	0.75	1.01	0.63
19	I do not enjoy reading non-medical literature or the arts^a^	6.3 (1.4)	0.45	0.58	0.37
**FACTOR 3: “STANDING/WALKING IN THE PATIENT's SHOES”**					
3	It is difficult for me to view things from my patients' perspectives^a^	5.2 (1.6)	0.60	0.81	0.45
6	Because people are different, it is difficult for me to see things from my patients' perspectives^a^	5.5 (1.5)	0.72	1.19	0.56
18	I do not allow myself to be influenced by strong personal bonds between my patients and their family members^a^	3.7 (1.7)	0.24	0.48	0.31

a *Responses were reverse-scored on these items; otherwise, items were scored directly (strongly disagree = 1, strongly agree = 7)*.

b *Factor loadings for the principal components analysis*.

c *Factor loadings for the confirmatory factor analysis*.

d *Item-total correlation Spearman's coefficient*.

**Table 4:** **Goodness of fit indexes of the correlated 3-factor model B of the Spanish JSE-HP version by gender, professional status, place of birth, studies, and residence**.

**Model**	**χ^2^**	***df***	**Ratio χ^2^/*df***	**CFI**	**TLI**	**RMSEA**	**SRMR**
**GENDER**							
Men	150	167	0.898	1.000	1.000	0.000	0.050
Women	170	167	1.018	0.998	0.998	0.007	0.056
**PROFESSIONAL STATUS**							
Physician	141	167	0.844	1.000	1.000	0.000	0.046
Physician-in-training	203	167	1.216	0.984	0.982	0.026	0.061
**PLACE OF BIRTH**							
Spain	137	167	0.822	1.000	1.000	0.000	0.051
Latin America	196	167	1.171	0.990	0.989	0.022	0.056
**PLACE OF STUDIES**							
Spain	131	167	0.782	1.000	1.000	0.000	0.052
Latin America	193	167	1.155	0.991	0.990	0.022	0.056
**RESIDENCE**							
Spain	143	167	0.856	1.000	1.000	0.000	0.045
Latin America	210	167	1.260	0.980	0.977	0.032	0.067

*χ^2^, Chi-square statistic; df, degrees of freedom; CFI, comparative fit index; TLI, Tucker-Lewis index; RMSEA, root mean square error of approximation; SRMR, standardized root mean square residual*.

### Group comparisons

Neither the entire sample, nor the sub-groups studied showed normal distribution empathy scores. Consequently, comparisons were made based on empathy global scores using non-parametric tests. The comparisons revealed statically significant differences (*p* < 0.001) among all the studied groups. The complete report of this analysis is shown in Table 5.

**Table 5:** **Comparisons of global score of the Spanish JSE-HP version according to the variables studied**.

**Characteristics**	**N (%)**	**Median**	**Mean**	**SD**
Global score (JSE-HP)	715	119	116	14
GENDER^a^^***^				
Men	332 (47)	117	114	15
Women	382 (54)	121	119	13
PROFESSIONAL STATUS^a^^***^				
Physician	311 (42)	117	114	13
Physician-in-training	422 (58)	120	118	15
SPECIALITY^b^^***^				
No speciality	73 (10)	117	115	14
Non-hospital speciality	243 (34)	120	118	13
Hospital speciality	252 (36)	120	117	14
Medical-Surgical speciality	48 (7)	119	117	14
Surgical speciality	65 (9)	109	108	17
Other	28 (4)	119	115	13
PLACE OF BIRTH^a^^***^				
Spain	351 (50)	122	121	11
Latin America	347 (50)	115	112	16
PLACE OF STUDIES^a^^***^				
Spain	363 (51)	122	121	11
Latin America	344 (49)	115	112	16
RESIDENCE^a^^***^				
Spain	480 (66)	121	120	12
Latin America	253 (35)	113	110	16
MIGRATORY CONDITION^b^^***^				
Spaniard living in Spain	351 (50)	122	121	11
Latin-American living in Spain	94 (14)	119	117	14
Latin-American living in Latin-America	253 (36)	113	110	16

SD, Standard deviation;

a U Mann-Withney test;

b Kruskal Wallis test;

*** *p < 0.001*.

Gender comparisons revealed that female physicians scored higher in empathetic interaction than male physicians. When participants were compared according to their professional status, physicians obtained lower global empathy scores than physicians-in-training. In both cases, the group differences were statistically significant (*p* < 0.001).

Statistically significant differences also appeared in speciality comparisons. Physicians qualified in surgical specialities obtained the lowest global scores. When this group was excluded from the group comparison, the differences were not statistically significant (*p* = 0.41). On the other hand, physicians qualified in non-hospital specialities obtained the highest global empathy global scores. This group included 240 family physicians, and 3 occupational physicians.

**Figure 1:**
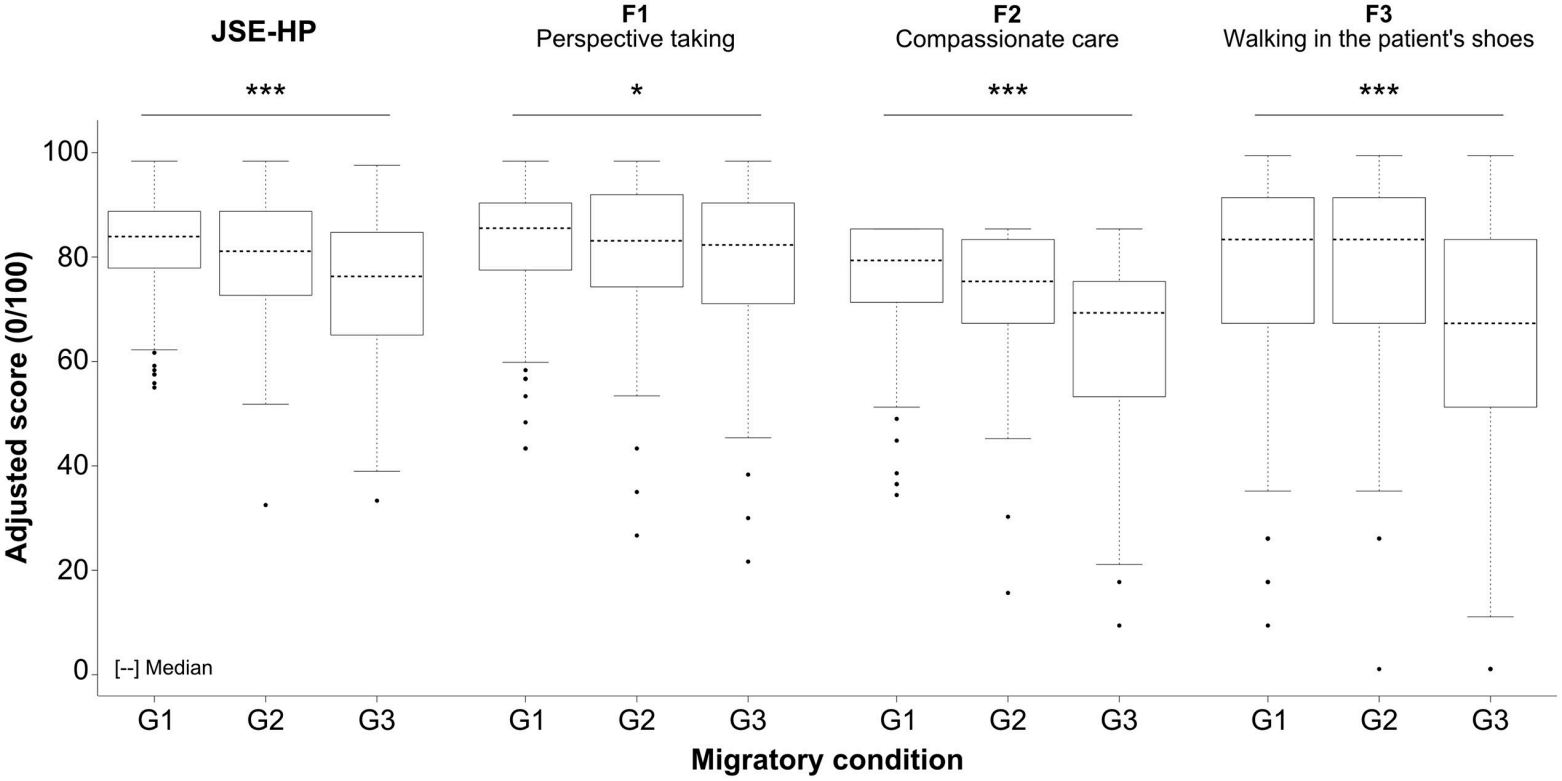
Comparative analysis of the 3-factor structure of the JSE among the Spanish physicians living in Spain (G1), the Latin-American physicians living in Spain (G2), and the Latin-American physicians living in Latin-America (G3); ^*^p < 0.05; ^***^p < 0.001.

Socio-demographic group comparisons revealed important cross-cultural differences (*p* < 0.001). The Spanish group obtained the highest global empathy scores, followed by the group of Latin American physicians with cross-cultural exchange experience in Spain. The group of physicians who had never left Latin America obtained the lowest global empathy scores. The comparison of the underlying factors of the JSE revealed significant differences in descending order of magnitude for “compassion care” (*p* < 0.001), “standing/walking in the patient's shoes” (*p* < 0.001), and “perspective taking” (*p* = 0.04), as can be observed in Figure 1.

## Discussion

The JSE was originally designed as a research tool to measure the development of empathy within the specific context of medical care and interaction with patients. Since it was first published in 2002, the JSE has been widely accepted at the international level. The first publications reported consisted mainly of exploratory studies with PCA (Alcorta-Garza et al., 2005; Di Lillo et al., 2009; Kataoka et al., 2009; Paro et al., 2012; Suh et al., 2012; Wen et al., 2013). However, in recent years there has been an increase in the number of publications including studies based on CFA. The authors of one of the latest articles (Hojat and Lanoue, 2014) use CFA to validate the JSE's psychometric properties. They also recommend the use of the CFA, and suggest maintenance of all the 20 items in the instrument, not only for goodness of fit of the 3-factor model, but also to obtain significant item-total correlations and substantial item discrimination effect size indexes for all items.

The results obtained from this study prove the stability of the JSE's most relevant characteristics: high reliability of the instrument, need for the inclusion of all the items and the existence of a 3-factor model composed by two main factors, one major cognitive and one emotional, and a third trivial factor. However, for research based on samples of Spanish and Latin American individuals, this study shows that it is preferable to include item 18 within the “standing/walking in the patient's shoes” factor, rather than within “compassionate care.” The differences observed for item 18 are in agreement with the findings of other authors whose works are not necessarily focused on the Spanish cultural context, but also on others. The study also reveals that the factors that make up the JSE follow an “oblique,” rather than “orthogonal” model. This difference is explained by the fact that empathy is understood as a cognitive-emotional unit where each of the factors influences the development of the remaining two, rather than as a sum of statistically independent units. Certain researchers (Magalhaes et al., 2011; Leombruni et al., 2014) and, more recently, authors themselves (Hojat and Lanoue, 2014), have pointed out the care that must be taken when approaching this topic.

Group comparisons yield differences that are consistent with those of previous studies, both for the categories of gender (Hojat et al., 2002; Alcorta-Garza et al., 2005; Kataoka et al., 2009; Magalhaes et al., 2011; Tavakol et al., 2011b; Suh et al., 2012; Shariat and Habibi, 2013; Wen et al., 2013; Leombruni et al., 2014) and speciality (Hojat et al., 2002; Suh et al., 2012). In agreement with the observations of other researchers, this study provides evidence of a positive association between the development of empathy and work in professional specialities involving greater roles in patient care (Tavakol et al., 2011a). In the case of students, variations may be explained by differences related to personality or emotional intelligence (Costa et al., 2014; Hojat et al., 2015), or by the influence of psycho-social factors (Hojat, 2006). With regard to practicing physicians, together with the differences mentioned above, the development of empathy might be influenced by professional burnout caused by exposure to adverse work environments (Almeida, 2002; Brazeau et al., 2010; Delgado-Bolton et al., 2015).

In the healthcare area, Latin America's complex economic, political, social, and cultural network poses a constant challenge (Cotlear et al., 2015). Added to professional limitations in disease treatment are inequities of access to the healthcare system, scarcity and misuse of resources, corruption in the sector and a high social demand. Even though there have been significant improvements in the sector (Atun et al., 2015), they are not consistent throughout the different regions and the challenges are hardly met. Proof of this is the deterioration in the physician-patient relationship, which is still far too common (Correa and Javier, 2011; Delgado-Bolton et al., 2015; San-Martín et al., 2016). The analysis of cultural variables carried out in this study proves the latency of this issue, while also providing clues about the importance of the role played by professional, cultural, and social surroundings in the development and improvement of medical empathy.

On the other hand, there are very important evidences in support of the validity and utility of the JSE in the context of patient care. The use of the JSE as indicator of this professional ability to predict clinical and patient outcomes in adult patients with diabetes was proved in two studies (Hojat et al., 2011b; Del Canale et al., 2012). In both cases an association between empathy in patient care and positive outcomes was confirmed. This is utterly important because the ultimate goal of medical education and all other health professions is to optimize clinical outcomes. In two studies, carried out with French physicians, a protective role of empathy for burnout (Lamothe et al., 2014), and a positive relationship between empathy and overall better clinical practice was demonstrated (Zenasni et al., 2012). Moreover, in Latin American and Spanish physicians-in-training with higher empathetic scoring in the JSE presented better and more effective learning abilities, and abilities toward inter-professional collaboration (San-Martín et al., 2016). In medical and healthcare education contexts, there are important evidences in support of the importance of the JSE (Hojat, 2016). Nevertheless, the authors are cautious and recognize that further research is needed to investigate the relationship between scores on the JSE and educational outcomes. Furthermore, large-scale research is also needed with national samples to develop national norm tables and cutoff scores for the JSE to identify low and high scorers in different populations and health professions students (Hojat and Gonnella, 2015).

In general, all these findings underlying the importance that empathy has in medical and healthcare education, in clinical practice, and in practitioners' health and welfare. Understanding the role of empathy is an issue with special relevance in geographical contexts where practitioners have to address daily social needs with scarce resources, as it happens in many public health institutions of Latin American countries.

## Author contributions

LV and AA undertook the translation and linguistic adaptation of the scale. MS and LV performed the statistical processing of data. LV was in charge of the study's overall design, coordination with the participating institutions, and drafting of the manuscript. RD, HR, and JS were in charge of the coordination with Spanish Healthcare institutions and LV, MS, and AA were in charge of the coordination with Latin American Healthcare institutions. All authors contributed to the presented work. All authors participated during the interpretation process of the results, and approved the final manuscript.

## Funding

This study was supported by the Rioja Salud Foundation (FRS), Spain.

### Conflict of interest statement

The authors declare that the research was conducted in the absence of any commercial or financial relationships that could be construed as a potential conflict of interest.
